# High-resistivity metal-oxide films through an interlayer of graphene grown directly on copper electrodes

**DOI:** 10.1007/s41127-017-0016-3

**Published:** 2018-02-06

**Authors:** Sieglinde M.-L. Pfaendler, Andrew J. Flewitt

**Affiliations:** Electrical Engineering Division, Department of Engineering, University of Cambridge, 9 J J Thomson Avenue, Cambridge, CB3 0FA UK

**Keywords:** Transparent metal oxides, Contacts, Channel conductivity, Stability, Barrier, Encapsulated, Low-temperature CVD graphene

## Abstract

Functional oxides are important materials for multiple applications in flexible and transparent electronics. Electrically contacting these oxides to form active channels is often challenging as they suffer significant alteration or instabilities when interfaced with metal electrodes. Here, we demonstrate a new scheme to electrically contact thin films of semiconducting zinc tin oxide (ZnSnO) that employs pre-patterned copper electrodes encapsulated by chemical-vapour-deposited graphene. Measurement of over more than 100 channels with varying geometry and nature of contact shows that the bulk resistivity of the ZnSnO channels with graphene/Cu composite is at least two orders of magnitude larger than the same films deposited directly on aluminium (Al) contacts. Moreover, the ZnSnO channels with Cu/graphene contacts showed nearly ohmic transport, in contrast to space-charge-limited conduction observed for other contacting schemes. Our results outline a new application of graphene in a step towards the development of alternative contacting strategies for oxide electronics.

**Electronic supplementary material:**

The online version of this article (doi:10.1007/s41127-017-0016-3) contains supplementary material, which is available to authorized users.

## Introduction

The electrical properties of the semiconducting oxide films can change dramatically in the presence of metallic electrodes. The interfacial composition of the oxide and metal thin film may be modified due to intermixing of the elements during fabrication, oxidation of the electrodes from air exposure prior to deposition of the functional oxide or post-fabrication diffusion of oxygen across the metal–oxide interface [1–3]. While suitable contact materials are already limited in number due to the work functions and large bandgaps of these metal oxides [2, 3], chemically inert electrical contacts are also critical to oxide electronics. This is because chemical modification to the oxide–contact interface can affect the conduction channel more adversely than merely adding a contact resistance, for example, via localized doping (localized inversion layer), interaction of the orbitals and completion of dangling bonds [4].

The chemical vapour deposition of graphene onto patterned thin films of copper [5–10] has been extensively researched in the context of electronic applications. In addition, the capability of graphene to protect the surface of the films against surface contamination, such as oxidation, moisture and adsorption, has been a subject of severe debate [6, 8, 9, 11–15]. The near impermeability [13–16] and structural resilience [11, 17] of graphene can prevent or mitigate the barrier diffusion processes, for example, out-diffusion of metal atoms or in-diffusion of foreign species when such a graphene-passivated copper film is physically combined with another material to form an electronic device [18]. This can naturally form a strategy to obtain non-invasive electrical contacts to a large class of metallic and semiconducting active/passive elements in electronics, although a systematic study towards integrating graphene-coated copper film as electrical contact to semiconducting oxides has not been carried out so far.

In this work, we report fabrication and electrical characterization of remote plasma sputter-deposited conductive ZnSnO thin films that are contacted by pre-patterned copper films with an interfacial coating of chemical-vapour-deposited (CVD) graphene. The key observation is that the bulk resistivity of the conductive channel is at least two orders of magnitude larger than when the same films are deposited directly on similarly patterned bare aluminium (Al) contacts. Moreover, the ZnSnO channels showed space-charge-limited transport for all contact types, except when the graphene interfacial layer was used. We discuss that the barrier properties of graphene at the copper surface allow superior preservation of the oxide channel by preventing, or at least reducing, the exchange of atomic species across the copper–ZnSnO interface.

## Sample description

**Fig. 1:**
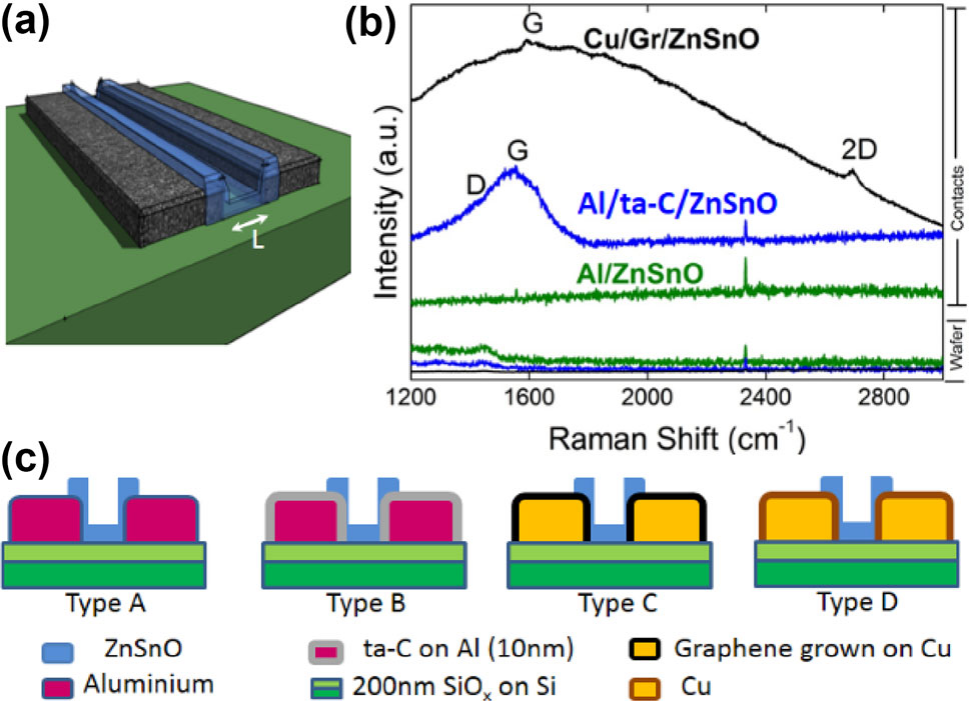
**a** 3D schematic of a fabricated device. The *green area* is the wafer, *black* the contact and *blue* the oxide, ZnSnO. **b** Raman spectra of the contact area. The distinct peaks at the G and 2D modes can be identified for the Gr/Cu contacts in spite of the broad background from the underlying copper film. **(c)** The four contacting schemes adopted in this work. The ZnSnO channel is deposited on bare aluminium films (Type A), aluminium films coated with amorphous carbon film (Type B), copper films coated with CVD grown graphene (Type C) and bare copper film (Type D)

Pairs of rectangular thin film contacts of equal width, *W*, 200 µm, and five different lengths, *L* (80, 40, 20, 8 and 4 µm), were patterned onto four silicon oxide wafers. These five patterns were replicated in batches at seven locations on each wafer (Supplementary Figure S1 is a location map of these batches). Subsequently, the zinc tin oxide was deposited into the pattern using a remote plasma deposition High Target Utilization System (HiTUS) [19–21], i.e. the metal oxide is only found bridging each of the pre-patterned pair of contacts. Figure 1a shows a three-dimensional schematic of the device structures investigated in this work.

Four contacting configurations were adopted for the ZnSnO channel; these are schematically shown in Fig. 1c. In wafer type A, the contacts consist of 100 nm films of thermally evaporated aluminium (Al), the most commonly used contact material for Zn-based thin film devices. In wafer type B, contacts consist of Al films with an additional protective layer of tetrahedral amorphous carbon, also known as diamond-like carbon or ta-C (10 nm), deposited onto the Al prior to lift-off using a filtered cathodic vacuum arc. In wafer type C, contact material consists of RF-magnetron-sputtered copper films, which were subsequently subjected to a standard process of chemical vapour deposition of graphene via decomposition of methane at elevated temperatures [5–7, 10]. This process results in an encapsulation of the contacts by mono-/few-layer graphene. Post-growth Raman spectroscopy (Fig. 1b) shows clear signatures of the characteristic G (1580 cm^−1^) and 2D (2760 cm^−1^) modes, in spite of the broad background from the underlying copper film. The graphitic backbone of ta-C encapsulation can also be seen in a broad peak around the G mode. Wafer type D consists of bare copper contacts which we did not subject to graphene encapsulation.

## Results and discussion

**Fig. 2:**
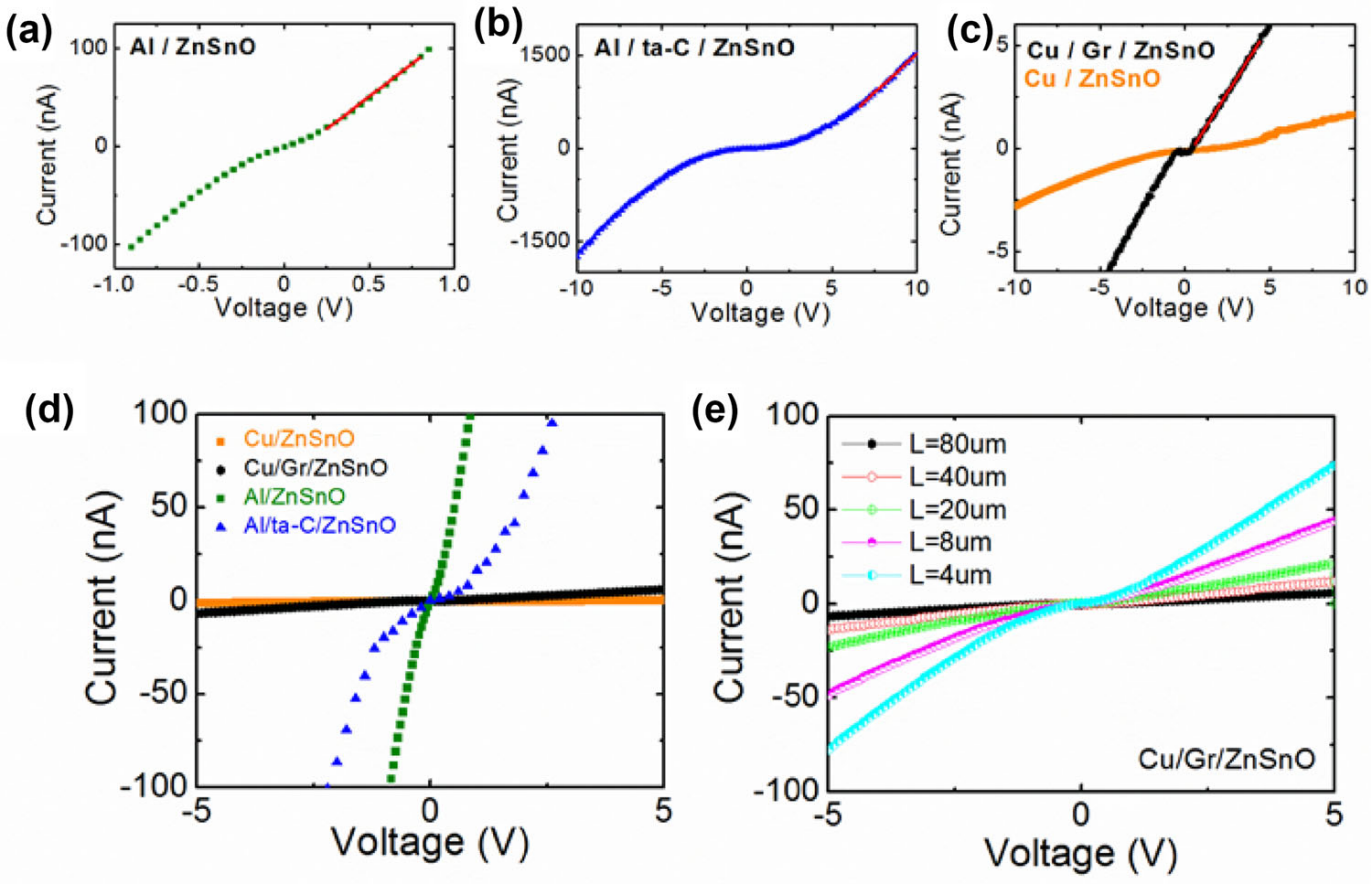
**a–c** Current–voltage characteristics for 80-μm-long device from the same location of the Al/ZnSnO (Type A), Al/a–C/ZnSnO (Type B) and Cu/Gr/ZnSnO (Type C), respectively. **d** The current–voltage characteristics of (a–c) plotted together on the same axis for comparison. **e** The current–voltage of a batch of devices of different lengths with Cu/Gr/ZnSnO contacts

Figure 2 shows a set of two-probe current (*I*)–voltage (*V*) characteristics of the ZnSnO channel for different contact types. The data shown in Fig. 2a–c were obtained in ZnSnO channels of length, *L* = 80 µm for each contact type. For types A, B and D, the *I*–*V* characteristics show nonlinear gap-like characteristics in all devices near *V* = 0 V, indicating formation of a barrier between the metallic contact and the ZnSnO channel. Such a barrier could be either a Schottky barrier due to band bending at the metal–semiconductor interface, or a physical tunnel barrier due to encapsulation, for example, due to ta-C in contact type B. In contrast, the *I*–*V* characteristic is remarkably linear for Cu/Gr/ZnSnO devices (Type C), although a gap of ~0.5–1 V, presumably due to an interfacial potential barrier, appears at around *V* = *0*. This is shown in greater detail in Fig. 2e. Nonetheless, the qualitative difference in the shape of the *I*–*V* traces implies the interfacial characteristics of the ZnSnO–Cu/graphene contact are very different from other contact types. The extent of nonlinearity at larger *V* was found to be configuration dependent and is discussed in the context of Fig. 5.

Apart from the qualitative nature of the *I*–*V* characteristics, another crucial observation is the difference in the magnitude of current between different contacting material combinations. As shown in Fig. 2, *I* increases to as high as 100 nA at *V* = 1 V for contact type A, which is >10 or >100 times that of the devices that had ta-C or graphene coatings on the metallic contacts, respectively, at the same applied voltage, namely *V* = 1 V. This naturally indicates that the Cu/Gr/ZnSnO devices are different from the Al/ZnSnO and Al/ta-C/ZnSnO devices in terms of the channel resistivity, contact resistance or both. To establish this, we have subsequently measured the two-probe resistance (*R*) for all devices/contact types at different channel length *L*, given by the separation between the pre-fabricated contact pair.

**Fig. 3:**
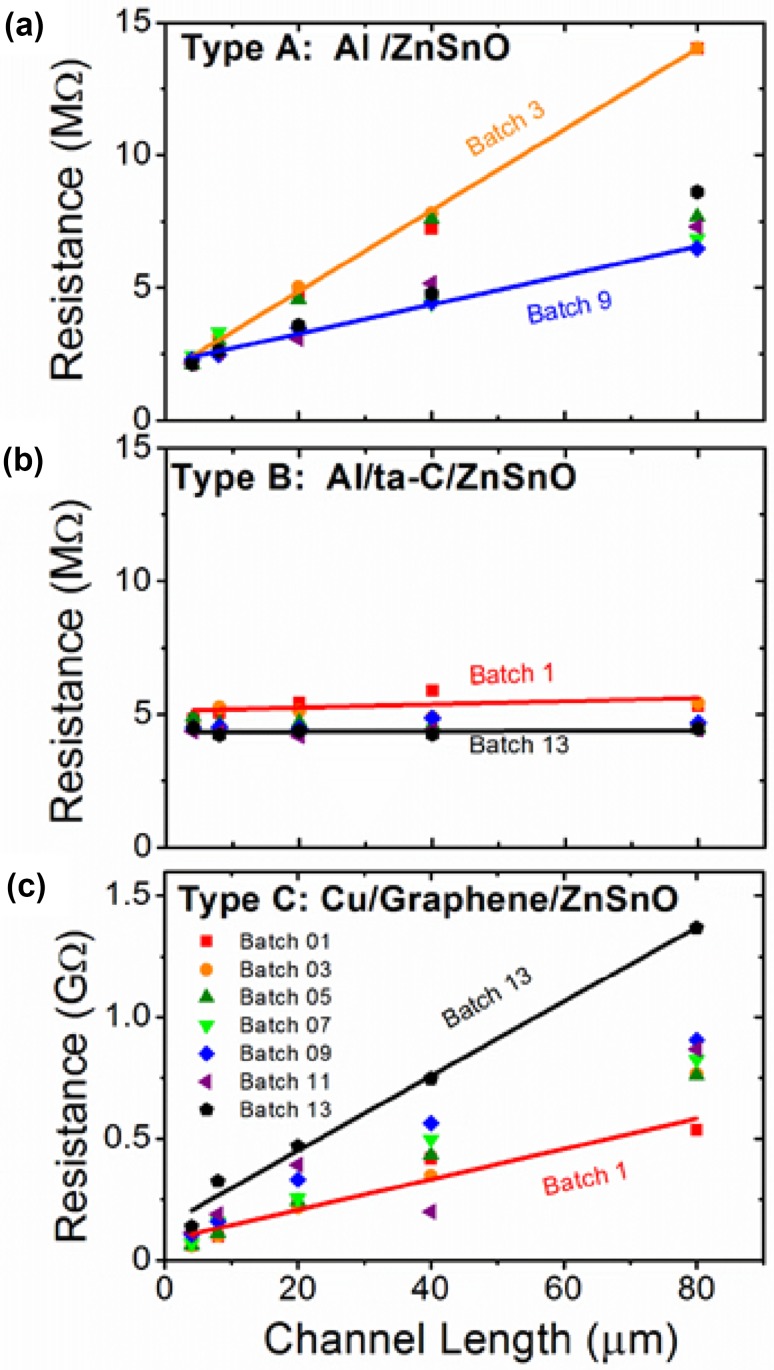
**a-c** Device resistance as a function of length for all the Al/ZnSnO, Al/ta-C/ZnSnO and Cu/Gr/ZnSnO devices, respectively. A batch of devices comprises of five devices of different lengths (4, 8, 20, 40 and 80μm). Seven batches for each contact type were measured. These are labelled Batch 1(*red square*), Batch 3 (*orange circle*), Batch 5 (*dark green upward pointing triangle*), Batch 7 (*light green point-down triangle*), Batch 9 (*blue diamond*), Batch 11 (*purple left-pointing triangle*) and Batch 13 (*black polygon*). The gradient and intercept of these graphs are used for Fig. 4. For the sake of clarity, only the linear fit for the steepest and most shallow batch is labelled with batch name and shown in the graph. A sketch of the orientation of device batches is available in Supplementary

Figure 3 indicates that *R* varies linearly with *L* for Al/ZnSnO (Fig. 3a) and Cu/Gr/ZnSnO (Fig. 3c) devices, albeit with very different slopes and *y*-intercepts, while the trend in Al/ta-C/ZnSnO was found to be weak and scattered. This behaviour was observed in all seven batches of each contact configuration. The key aspect of Fig. 3 is that in spite of the linear dependence of *R* on *L*, the oxide channels in the Cu/Gr/ZnSnO devices have a far larger *resistivity* than in the Al/ZnSnO devices. This is a surprising result because the resistivity of the material is generally considered to be an intrinsic property and one would expect it to be constant since all three wafers have identical channel material sputter deposited at the same time. *Notably, for the Cu/ZnSnO devices (type D), the majority did not conduct*, and the conduction was so poor in the remaining samples that it was not possible to fit a straight line to the *I*–*V* nor discern a trend in resistance per unit length (orange trace, Fig. 2c). Copper is a poor contact for zinc oxide as the electron affinity of Cu is 5.22 eV, and the electron affinity of this ZnSnO will approximately be that of ZnO (4.35 eV and bandgap of 3.37 eV) [22]. Thus, one is looking at a Schottky barrier of close to 90 meV at the copper–ZnSnO interface, which will make very poor contact. In addition to this, we speculate the oxidation of copper surface upon deposition of the ZnSnO oxide layer into a creation of an insulating oxide at the interface creating a further conduction barrier.

For a quantitative analysis, the measured *R* in our devices can be written as,

$$ R = R_{\text{contacts}} + R_{\text{channel}} = \rho_{\text{contacts}} /{\text{Wt}} + \rho_{\text{channel}} L/{\text{Wt}} $$

where $\rho_{\text{contacts}}$ and $\rho_{\text{channel}}$ are the *specific contact resistance* and *bulk resistivity* of the channel, respectively. Thus, the slope of the *R* versus *L* plot provides the channel resistivity, $\rho_{\text{channel}}$, while the contact resistance $R_{\text{contacts}}$ is the intercept of the linear fit (when *L* = 0 µm) on the *y*-axis. The width, *W* (=200 µm), and the thickness, *t* (=90 nm), were kept constant for all samples.

**Fig. 4:**
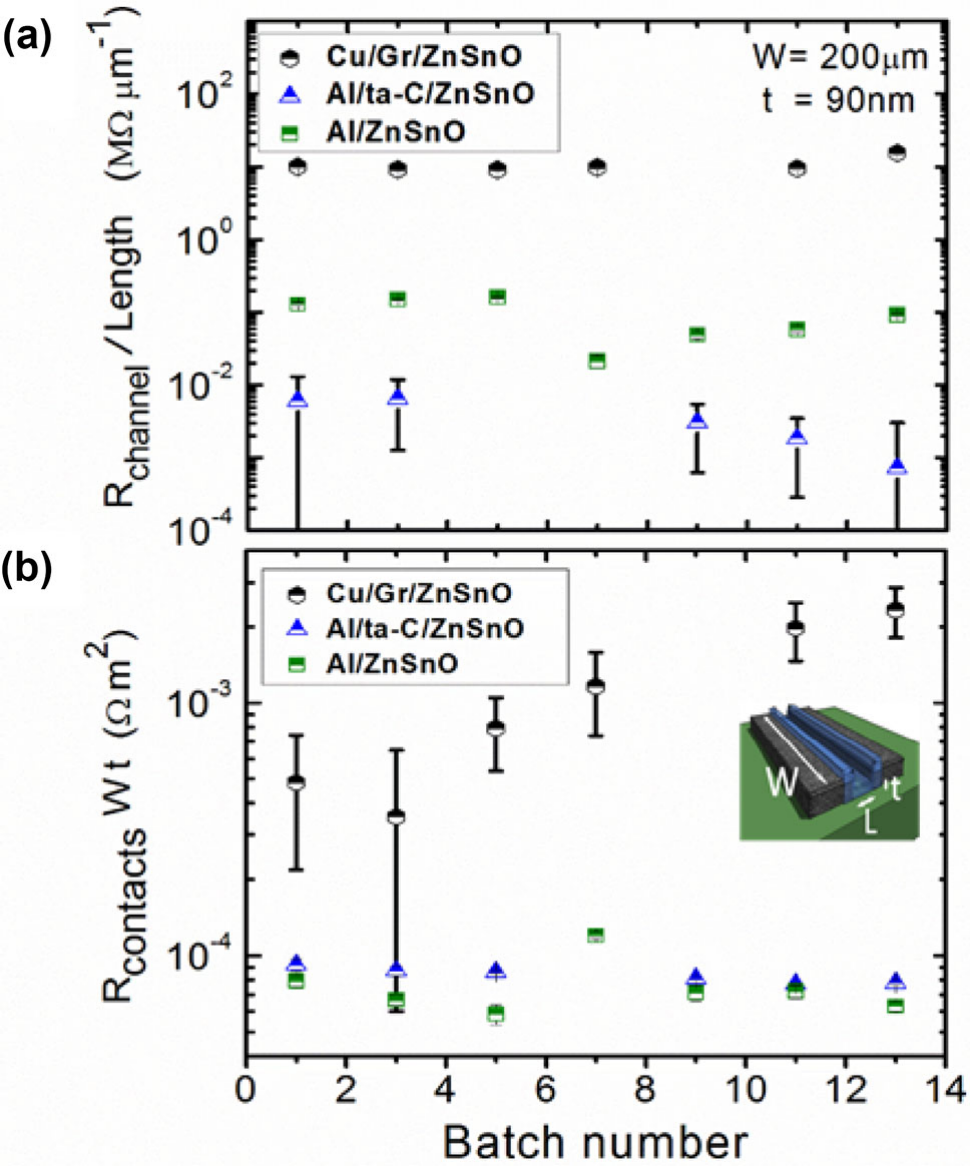
**a** Channel resistance per unit length for all the batches on the Al/ZnSnO (*green squares*), Al/ta-C/ZnSnO (*blue triangles*) and Cu/Gr/ZnSnO (*black hexagons*). **b** Specific contact resistance (*R*_contacts_ W t) [also commonly known as *ρ*_Contacts_] for each batch is 10 to 100*x* larger for Cu/Gr/ZnSnO than for the Al/ZnSnO. *Inset* in (**b**) of the device indicates the location of the channel width, *W*, length, *L*, and film thickness, *t*

Figure 4 summarizes the key result of this work, where we have shown the channel resistivity and specific contact resistance from the *R*–*L* plots of the three devices architectures. Figure 4a shows the channel resistance per unit length ($= \rho_{\text{channel}} /{\text{Wt}}$), which shows a batch-to-batch agreement, indicating the ZnSnO deposition has been uniform over the entire wafer area, for all contact configurations. The striking observation, however, is that the *resistance per unit length* of the device with the graphene interlayer (type C: Cu/Gr/ZnSnO) is larger by ~2 orders of magnitude than that of the Al-contacted device (type A: Al/ZnSnO) and nearly 3–4 orders of magnitude than that of Type B (Al/ta-C/ZnSnO) devices. This implies a fundamental difference in the *bulk properties of the channel material when metal contacts are encapsulated with graphene.* While the channel resistivity of Al/ta-C/ZnSnO devices is even lower than of the Al/ZnSnO devices, a significantly larger contact resistance (see Fig. 4b) makes estimation of the channel resistivity in the Al/ta-C/ZnSnO devices somewhat inaccurate.

Figure 4b shows the specific contact resistance extracted from the *R* versus *L* plot for all devices. The contact resistance in Al/ta-C/ZnSnO is about twice that of Al/ZnSnO, which can be understood as the contribution from the ta-C interlayer in addition to the Al/ta-C interface. However, the contact resistance in the Cu/Gr/ZnSnO devices is ~10–100 times larger, suggesting that high contact resistance and large bulk resistivity in these devices need to be considered from a common conceptual platform.

For a microscopic description of the interfacial processes in the devices with graphene interlayer, we note that graphene was initially chosen as it was deemed to be a physically strong and impermeable barrier to gases including oxygen and helium [17]. However, the use of chemical-vapour-deposited graphene to provide oxidation protection to metal surfaces, such as Cu and Cu/Ni alloys [23], and as an oxygen barrier for a gate dielectric [24], met with limited success. Leakage at grain boundaries and other defect sites, and diffusion of oxygen radicals in extreme conditions, such under UV exposure, through the grain boundaries [25] have been observed. To counter this, recent studies have found that such graphene defects can act as nucleation centres for metals consequently deposited on them and help make the graphene defect impermeable again [13]. In our case, ZnSn is remotely sputtered onto the graphene in an oxidizing atmosphere, such that there is a chance that ZnSn could nucleate at the defects and block them. While this may not lead to total impermeability, one expects graphene to impose some resistance to oxygen migration across the interface in the Cu/Gr/ZnSnO devices. This can help not only in maintaining the oxygen stoichiometry of the as-deposited oxide channel, but also in mitigating intermixing of the contact metal and channel during sputtering (fabrication) [26]. Suppressing out-diffusion of oxygen reduces oxygen vacancy concentration in the channel and allows preserving the bulk resistivity to that of the intrinsic oxide channel [27, 28].

While the microscopic origin of the large contact resistance in the Cu/Gr/ZnSnO devices is not addressed in these experiments, several possibilities are suggested: a modification of the work function by the underlying metal [29], or a lower density of states of the strongly insulating channel at the Fermi energy of the contact, and/or the large out-of-plane resistivity of graphene due to poor hybridization of the graphene wave function with the oxide [22, 30]. Nonetheless, despite the different possible roles of the integrated graphene layers: as an oxygen barrier to reduce diffusion of oxygen between the Cu contact and the oxide film, ensuring long-term device stability, and/or as a protective interlayer during fabrication, the key result from these experiments is that the graphene reliably had a drastic effect on the bulk electrical resistivity of the channel material.

It is indeed surprising that different contacting strategies can affect bulk channel resistivity even for channels as long as 80 µm. While we do not understand the specific mechanism at this point, we present various alternate conjectures regarding this observation. Firstly, while oxygen diffusion over such large distances at room temperature appears unlikely [31], the diffusivity of oxygen ions in oxide is known to increase by several orders of magnitude even with modest rise in temperature [32–36]. The vacancies are likely to be created in the channel through out-diffusion of oxygen ions into the aluminium contacts. An alternative or additional conjecture is that Al is a dopant for ZnSnO, and while a remote sputtering deposition method was used (i.e. the sample is not directly in the plasma), it is possible that energetic atoms reaching the surface during the initial creation of the ZnSnO cause secondary sputtering of the contact material (Al) onto the entire channel area until sufficiently buried. Graphene continues to be known for having a very low sputter yield and therefore may prevent intermixing of the contact material during the subsequent oxide deposition [11], which would also explain the high resistivity of the Cu/Gr/ZnSnO channels. A further alternative is Cu contamination of the substrate in graphene CVD (if grown at high vapour pressure of Cu at the growth temperature) and instability of SiO_2_ at high temperatures (~1000°C) which could lead to the formation of silicides. In such a case, the change in resistivity could be due to Cu scattered onto the substrate during the graphene CVD process, altering the doping of the ZnSnO. We used a lower-temperature growth recipe for the CVD growth of graphene to reduce this possibility.

**Fig. 5:**
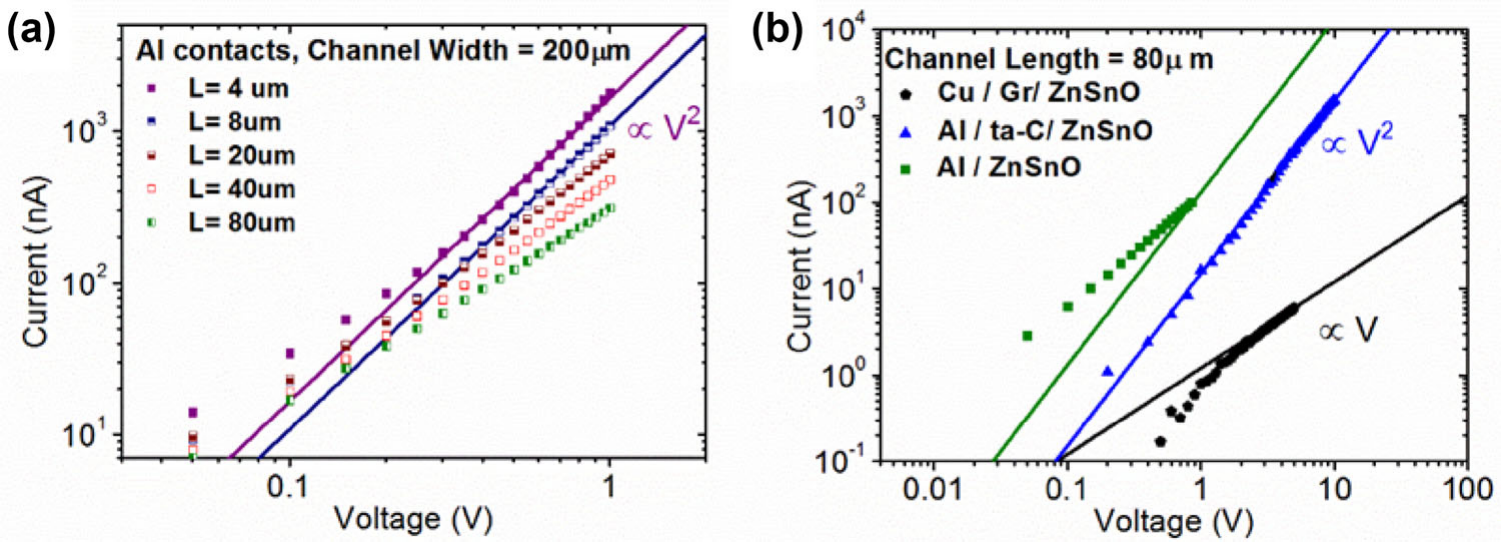
**a** Log (current) versus log (voltage) for all device lengths (4, 8, 20, 40, 80μm) demonstrating the *∝V*^*2*^ dependence for the Al contacts. **b** Log (current) versus log (voltage) of the curves in (a–c). Graphene covered sample experiences *I∝V*

A closer inspection of the *I*–*V* characteristics in the large current regime reveals another striking effect of graphene encapsulation of the metal contacts. The log(*I*)–log(*V*) characteristics of the Al/ZnSnO devices at five different lengths of the oxide channel are shown in Fig. 5a. For short channels (*L* = 8 μm and 4 μm), the current is proportional to *V*^2^, but this relationship becomes weaker for longer channel lengths. This is a characteristic feature of the Mott–Gurney law [37] (Fig. 5a), where *I∝V*^*2*^/*L*^3^, suggesting that the transport is limited by a space-charge region, particularly near the source contact–ZnSnO interface. Similar *I*–*V* characteristics were observed in Al/ta-C/ZnSnO devices as well (Fig. 5b), although at a longer channel length, trap-assisted Mott–Gurney transport reduces the bias exponent to slightly below two in the Al/ZnSnO devices. However, the channel of same length on graphene (Cu/Gr/ZnSnO) shows linear *I*–*V* characteristics even at large *V* ~5 V. In fact, we observed *I* *∝* *V* at large voltages even in shorter channels of Cu/Gr/ZnSnO devices, suggesting that when graphene separates the oxide channel from the metal (copper) contact, the charge injection from the contact fails to drive the channel out of charge neutrality. While this could relate to larger metal to oxide interfacial resistance in these devices, a space-charge-free interface even at large operating voltages may be desirable in device operations.

## Conclusions

In conclusion, different carbon-based strategies to achieve stable metal contacts to thin films of transparent metal oxides are presented. This work with sputtered ZnSnO films reveals that the addition of a graphene interfacial layer had a drastic effect on the bulk electrical properties of the oxide material (resistance per unit length and current–voltage characteristics) as consistently observed in a significant sample size of over hundred devices. The interlayer provides a solution that allows long-lasting control of the resistivity of the oxide channel. Recently developed techniques for low-temperature CVD of graphene may facilitate integration of such graphene-coated electrodes with flexible substrates and have the potential to influence device contacting strategies.

## Methods

### Patterning

Devices were patterned using conventional photolithography, deposition and solvent lift-off process employing an image reversal “Microposit AZ3516” photoresist and an EVG Mask Aligner, using the layout depicted in Supplementary Figure S1.

### Contacts metallisation

100 ± 5 nm of Al was evaporated using an Edwards 306 thermal evaporator and then lifted off using a three-stage rinse in an ultrasonic bath using acetone, isopropanol and then de-ionized water.

Cu contacts were deposited onto the patterned wafer using RF-magnetron sputtering of a Cu foil Alfa Aesar (99.999%) target.

For samples with graphene, the Cu was deposited in the aforementioned sputtering deposition; however, samples were taken through the lift-off process prior to graphene growth in the customized cold-wall reactor “Black Magic 3” Chemical Vapour Deposition System designed by AIXTRON. The sample patterned with Cu was heated and annealed in H_2_ ~4 mbar 210 sccm at 900 °C, followed by exposure to benzene for 30 min before cooling *in vacu*o [5]. Our graphene growth process is as described in the following references [5–7].

For samples with ta-C, the ta-C was deposited using a custom-built filtered cathodic vacuum arc (FCVA) system. These were confirmed to be 630 ± 10 nm using a Veeco DektakV surface profilometer using 2 mg of force.

### Remotely sputtered ZnSnO

Sputtering of the channel material was performed with a Remote Plasma, High Target Utilization Sputtering System (HiTUS), Model S500 (Side-arm configuration), designed by Plasma Quest. A metal ZnSn target was sputtered and oxidation of the channel material occurred during sputter deposition through a continual shower of oxygen placed in between the sample and the target. The percentage of tin relative to zinc after deposition is 8 as measured by X-ray photoemission spectroscopy [19]. The main advantage of this system is that the charged plasma is directed away from the sample such that the damage that would normally occur to carbon-based layers in traditional systems where the sample sits in the plasma is reduced [38]. Furthermore, in the HiTUS system, unlike in conventional RF-magnetron sputtering, the plasma density and energy of the plasma can be decoupled for a gentle (in terms of bombardment and temperature) yet quick deposition, in this case 4 min and 30 °C. The material here was confirmed to be amorphous using X-ray diffraction [19]. A piece of silicon with a native oxide (~2 nm) was placed with the samples during deposition. Thickness was measured using a Gaertner optical ellipsometer and confirmed to be 90 ± 5 nm. Surface profilometry of the patterned devices agreed with this value (using the aforementioned surface profilometer).

The resistivity and conduction mechanisms of ZnO-based materials are often attributed to the mobility of charge carriers from oxygen vacancies and can be modified by external influences such as moisture or light [39–45]. One approach to modifying the dominant conduction mechanism is by adding a third or fourth element in much higher proportions than is typical of semiconductor doping, such as In, Ga, Hf or Sn [44–47]. In many cases, the additive elements can be the majority species. Even so, the metal oxides remain sensitive to oxygen content during fabrication, to within a fraction of a per cent. The films presented here are made of zinc tin oxide (ZnSnO), where the main purpose of the tin in this study is to ensure that the layer is amorphous and thus encourage uniformity across the wafer, as verified previously [19].

The oxide deposition was performed for *all* wafers simultaneously, and they were all placed at the same distance from the centre of the rotating sample stage, ensuring thickness and composition uniformity. Thickness uniformity was confirmed by experiment (unpublished) in a set of test depositions placed at different locations on the sample stage. Thickness was measured by surface ellipsometry and surface profilometry. Composition uniformity was confirmed [19].

### Handling precautions

In practice, if the individual components of a multi-layer device, such as a transistor, are developed to have certain characteristics independently (such as resistivity or breakdown voltage), they need to be re-optimized to account for interaction of the materials with each other as well as the various fabrication conditions of each step, particularly heat treatment steps, or certain etchants including acidic water in the case of materials containing a high proportion of ZnO. Consistency during fabrication of these devices was respected. For example, the time taken to rinse and dry each sample was kept consistent.

### Raman

Raman was used to verify the presence of graphene on contacts after patterning and confirm the absence of carbon in between devices that would have created an electrical short had it been present.

Raman measurements were performed using a Renishaw Raman InVia Microscope with 532-nm laser excitation and using a 100× objective which gives a spot diameter of ~1 μm.

Figure 1b shows Raman spectra measured in different locations on the sample wafers following complete device fabrication. The spectra on the contacts of the Al/ta-C/ZnSnO show the broad, combined D and G peaks expected for ta-C, while no such peaks are observed on the Al/ZnSnO. For the Cu/Gr/ZnSnO contacts, 2D (~2700 cm^−1^) and G (~1600 cm^−1^) peaks confirm the presence of a graphene coating despite the large background signal associated with Cu photoluminescence when performing Raman using laser excitation of 532-nm wavelength.

## Electronic supplementary material

Below is the link to the electronic supplementary material.

Supplementary material 1 (DOCX 123 kb)
