# Supplementary material for: High-resistivity metal-oxide films through an interlayer of graphene grown directly on copper electrodes
Source: Graphene Technol. 2018 Feb 6;3(1):11–8. doi: 10.1007/s41127-017-0016-3 (PMC6951820; doi:10.1007/s41127-017-0016-3)
Supplement: Supplementary file 1 — Supplementary material 1 (DOCX 123 kb) [file 41127_2017_16_MOESM1_ESM.docx]

SUPPLEMENTAL INFORMATION:

**“**High resistivity Metal-oxide Films through an Interlayer of Graphene Grown Directly on Copper Electrodes**”**

By Sieglinde M.-L. Pfaendler* *et al.*

Electrical Engineering Division, Department of Engineering, University of Cambridge, 9 J J Thomson Avenue, Cambridge, CB3 0FA, UK

*sieglinde.pfaendler@cantab.net

Below is an enlarged figure of device configuration (inset of Figure3b of the article named above).


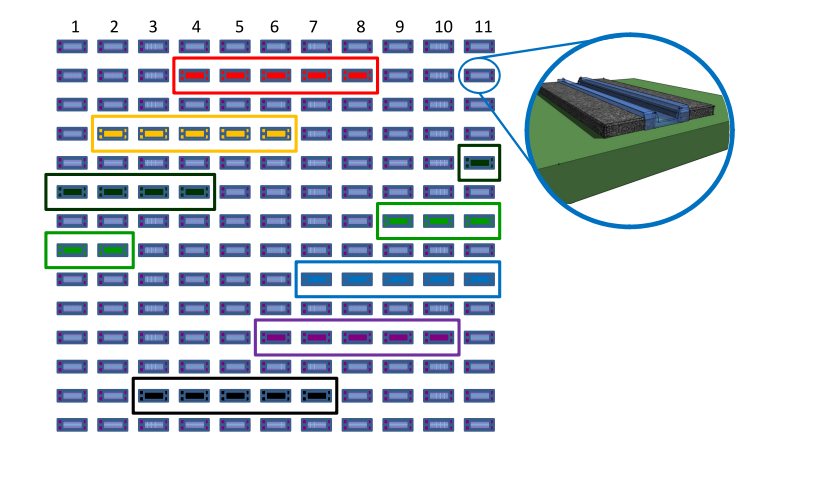


Figure S1 Top-view Layout of devices on a wafer. The batches outlined and colored are the ones presented in this letter. The locations are color coded using the color scheme from Figure 3 (Red, Orange, Dark-green, Green, Blue, Purple and Black representing Batch 1 to 7 respectively. Each batch outlined by the colored rectangle contains 5 devices each of a different length (80, 40, 20, 8 and 4μm). The bubble contains the 3D schematic of a device.
